# Differences in Real-world Functioning Between Adoptees With High or Low Risk for Schizophrenia Spectrum Disorders—The Finnish Adoptive Family Study of Schizophrenia

**DOI:** 10.1093/schizbullopen/sgaf011

**Published:** 2025-05-26

**Authors:** Matilda Raiskila, Helinä Hakko, Karl-Erik Wahlberg, Sami Räsänen

**Affiliations:** University of Oulu, Faculty of Medicine, Research Unit of Clinical Medicine, Psychiatry, Peltolantie 17, PT1, FIN-90220 Oulu, Finland; Department of Psychiatry, Oulu University Hospital, Peltolantie 17, PT1, FIN-90220 Oulu, Finland; University of Oulu, Faculty of Medicine, Research Unit of Clinical Medicine, Psychiatry, Peltolantie 17, PT1, FIN-90220 Oulu, Finland; University of Oulu, Faculty of Medicine, Research Unit of Clinical Medicine, Psychiatry, Peltolantie 17, PT1, FIN-90220 Oulu, Finland; Department of Psychiatry, Oulu University Hospital, Peltolantie 17, PT1, FIN-90220 Oulu, Finland

**Keywords:** genetic vulnerability, functional performance, adoptive family functioning, high risk for psychosis, schizotypal traits

## Abstract

**Background and Hypothesis:**

Schizophrenia spectrum disorders are known to affect the patient’s functional performance. The functioning of those who are at familial risk for these disorders is less well understood. In this study, we compared the real-world functioning of adoptees with a genetic high-risk (HR) for schizophrenia spectrum disorders with adoptees without this risk (low-risk, LR). We hypothesized that the HR-group would have more difficulties in real-world functioning compared to the LR-group.

**Study Design:**

The data were based on the Finnish Adoptive Family Study of Schizophrenia project. The study sample included 127 HR- and 130 LR-adoptees. An interview-based method, Strauss–Carpenter Level of Function (SCLF)-scale, was used to measure functional performance in a setup of adoptees living in comparable adoptive families. The SCLF-scale comprises domains for function, symptoms, social contacts, and work. The Structured Interview of Schizotypy was utilized in assessments of possible schizotypal traits of the HR- and LR-adoptees.

**Study Results:**

No significant differences in the real-world functioning in total scores or scores of any SCLF domains were observed between HR- and LR-adoptees. Of single SCLF items, the HR-adoptees were characterized as being less likely to have achieved formal education and the LR-adoptees needed more help with their own basic needs.

**Conclusions:**

No differences were found in the real-world functioning between HR- and LR-populations. This indicates that the real-world functioning does not express one’s genetic risk for schizophrenia spectrum disorders. Our findings highlight the importance of considering environmental factors when comparing genetically different groups.

## Introduction

Functional capacity can be defined as a person’s ability to accomplish daily tasks in their own environment in a self-satisfying way.^1,2^ While functional capacity describes what a person is able to do, real-world functioning describes what the person actually does.^3^ Functional capacity does not always directly correlate with real-world functioning, since many factors, such as lack of confidence and self-efficacy, as well as different motivational and environmental issues can affect it.^2,4^

The real-world functional outcome of patients with schizophrenia is often poor. Deficits can be seen, for example, in independent living skills, maintaining employment, the ability to fulfill basic social roles in the community as well as in verbal and nonverbal social skills.^5,6^ This deterioration is related to symptoms of the disorder, changes in functional capacity and in neuropsychological performance, such as impairment in cognitive skills.^2^ Deficiencies in function have also been stated to be potential first signs of the onset of the disorder.^7,8^ This has been observed as functionality changes in the prodromal phases of schizophrenia.

The functional deficits manifesting during different phases of schizophrenia have also been shown to be disproportionately prevalent among first-degree relatives of individuals with schizophrenia.^9–12^ For example, people at genetically high-risk of developing schizophrenia have shown to have more difficulties in social functioning compared to those without schizophrenia in their families. It has been shown that they struggle in interactions with peers and people of the opposite sex, lack initiative and motivation, and engage in fewer hobbies and pastimes in their free time.^12–15^ First-degree relatives of patients with schizophrenia also seem to have similar, although not as severe, cognitive deficits as the persons with the disorder.^14,15^ Additionally, people with a higher genetical predisposition to schizophrenia spectrum disorders have a high prevalence to autistic symptoms.^16^ Difficulties in functioning among young relatives of patients with schizophrenia may indicate a higher risk for developing schizophrenia later in life.^11,17^

When examining functioning, persons with schizotypal personality disorder should also be taken into consideration, since the risk for schizotypy seems to be increased in relatives of patients with schizophrenia.^18^ Schizotypy is defined as a group of personality traits that correlate with a higher liability for schizophrenia.^19–21^ These traits are qualitatively similar to some schizophrenia symptoms. For example, abnormalities in social functioning are a common feature for schizotypal personality.^22^ Despite this, schizotypy does not seem to be associated with general impairment in all the different domains of functioning.^23^

Thus far, the research concerning the functional outcome of people with a high genetic risk for schizophrenia is sparse and has focused almost entirely on the functional capacity of exposed persons. A very limited number of studies have been published in which overall real-world functioning has been the main interest of study. Additionally, previous studies have mainly been conducted in research settings, where the possible impact of upbringing and associated environmental factors during childhood and adolescence could not have been controlled in the statistical analyses.

### Aims of the Study

Using an adoption study design, we were able to assess the global real-world functioning of persons with a genetically high risk (HR) for schizophrenia spectrum disorders in comparison to adoptees with a low risk (LR) for these disorders. The adoption study design ensured that the adoptive family rearing environments of HR- and LR-study groups of the adoptees were matched to be comparable to each other. The aim of this study was to compare structurally measured real-world functioning between these study groups using the Strauss–Carpenter Level of Function (SCLF)-scale. Schizotypal traits of HR- and LR-adoptees, as assessed with the Structured Interview of Schizotypy (SIS)-research instrument, were also analyzed in relation to real-world functioning of the adoptees. Based on the comparatively small body of research on the subject, which indicates that individuals at HR for schizophrenia have more deficits in functioning and in cognitive performance, we hypothesize that the HR-population has more deficiencies in global real-world functioning.^9–11,24^ Our study complements research-based knowledge of the relation between real-world functioning and genetic liability for schizophrenia spectrum disorders.

## Material and Methods

### Study Population

The study population is based on the Finnish Adoptive Family Study of Schizophrenia.^25,26^ Adopted offspring of women, who have been hospitalized in Finnish psychiatric hospitals at least once between 1960 and 1979 due to schizophrenia or paranoid psychosis are compared to adoptees of biological mothers who had no psychiatric disorder or who had a psychiatric disorder other than schizophrenia spectrum disorder. The selection of biological mothers was conducted in 2 phases. Primary, mothers with affective or reactive psychosis were excluded and only mothers with schizophrenia or paranoid psychosis were included. In the second phase, the remaining biological mothers´ diagnoses were verified according to the DSM-III-R criteria (The American Psychiatric Association, 1987). Based on the categories by Kendler et al.,^27^ the following diagnoses of the broad schizophrenia spectrum were included: schizophrenia, schizotypal, schizoid, paranoid and avoidant personality disorder, schizoaffective, schizophreniform, and delusional personality disorder, psychotic disorder not otherwise specified and bipolar and depressive disorders with psychotic features. After the secondary diagnostic phase, a concentrated sample was formed, which also included 11 (6.5%) affective psychosis. Following diagnoses were verified: schizophrenia (*n* = 129, 75.9%), schizoaffective disorder (*n* = 6, 3.5%), schizophreniform disorder (*n* = 10, 5.9%), schizoid personality disorder (*n* = 2, 1.2%), delusional disorder (*n* = 5, 2.9%), psychosis NOS (*n* = 4, 2.4%), affective psychosis (*n* = 11, 6.5%) and non-psychotic, nonspectrum (*n* = 3, 1.8%). For verification of the diagnoses, health care records were reviewed, and personal research interviews were arranged for all index and comparison (control) mothers. Also, for all study subjects, Finnish national computer registers were examined in order to find information about causes of death, possible hospital discharges, diagnoses that justified disability pension, sick leaves, prescriptions for medications, and information about criminality.^28^ Adoptees that were adopted by a relative, adopted after the age of 4 years, or adopted to another country were not included in the study population. The adoptees of biological mothers with schizophrenia spectrum disorders were referred to as genetically HR adoptees and those with biological mothers without a schizophrenia spectrum diagnosis were referred to as low-risk (LR) adoptees. A more detailed description of the design and different procedures of the Finnish Adoptive Family Study of Schizophrenia can be found in earlier literature.^25,26,28,29^

The adoptive families of both HR- and LR-adoptees were matched by socioeconomic factors and family structure, as well as the age and sex of the adoptee and adoptive parents and the age of the adoptee at time of their adoption. Global Family Ratings (GFRs) were used^30^ to evaluate the functioning of adoptive families. Global Family Ratings are based on semi-structured interviews of individuals, couples, and families combined with observations made by the interviewer.

**Figure 1.**
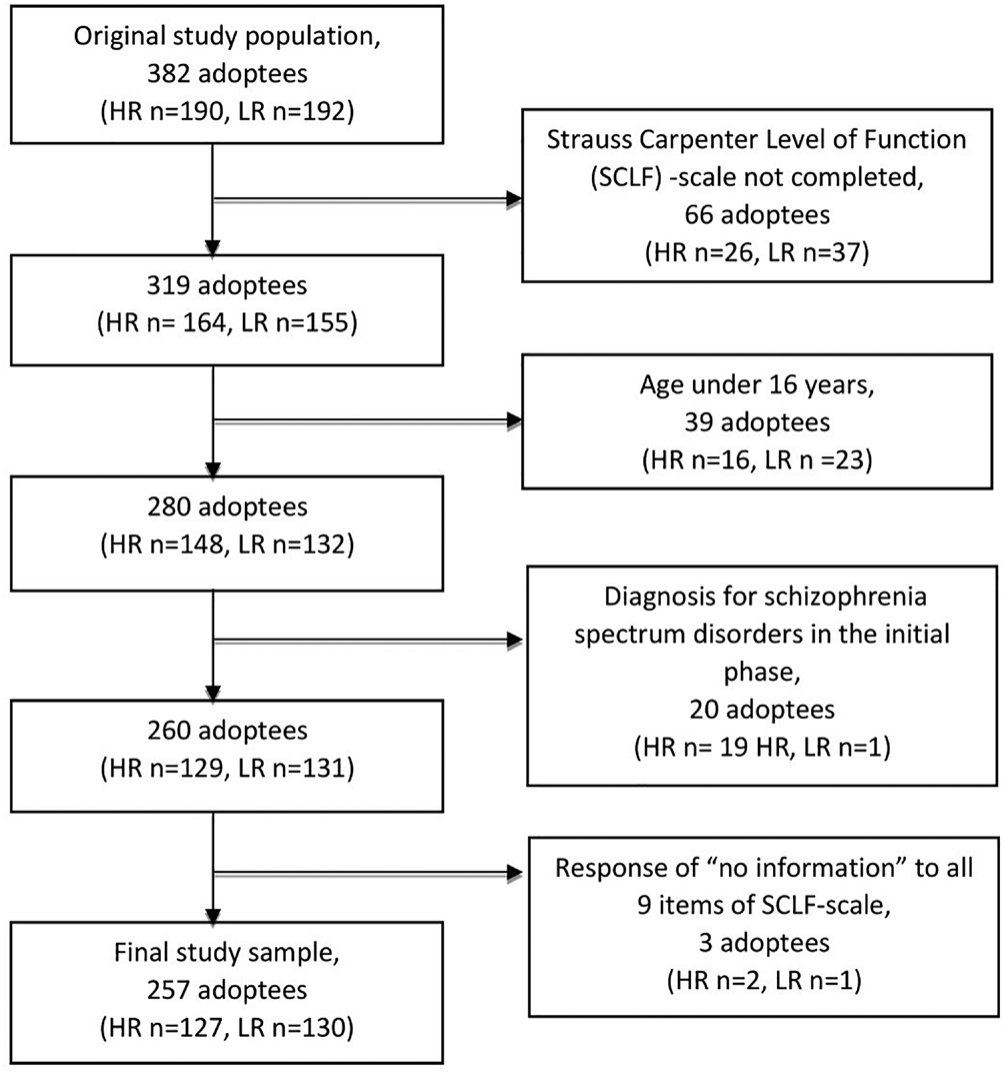
Sample Selection for the Current Study.

The initial study population consists of 190 HR and 192 LR-adoptees. In this study, we only included adoptees of working age (between 16 and 64 years), since the Strauss–Carpenter Level of Functioning Scale contains questions concerning working history. We also excluded from the analyses those adoptees who already at the initial phase of the study were diagnosed as having schizophrenia spectrum disorder, because these disorders are known to affect a person’s overall functioning. The final sample of the current study, of whom the SCLF was available, consisted of 257 adoptees (127 HR, 130 LR). The sample selection process for the present study is visualized in Figure 1.

### Levels of Functioning by Strauss–Carpenter

The real-world functioning of the HR- and LR-adoptees was assessed at the initial phase of the data collection using the Strauss–Carpenter Level of Function (SCLF)-scale. This is a semistructured interviewer-administered scale that has been developed and used for the evaluation of the functioning of patients with schizophrenia.^31–33^ The SCLF-scale consists of 9 items that fall into 4 domains, these being (1) function (3 items; ability to meet own basic needs, fullness of life and overall level of function), (2) symptomatology (2 items; absence of symptoms and duration of non-hospitalization for psychiatric disorder), (3) social contacts (2 items; frequency and quality of social relations) and (4) work (2 items; quality and quantity of useful work). Each item from all domains is scored from 0 to 4 on a Likert-type scale, with a higher score indicating better functioning.^34^ Domain scores are calculated as the mean scores for items within each domain. The total score of the scale is analyzed as a sum and as a mean of scores for all 9 items of the scale. The scale takes approximately 20 min to complete.

### Assessment of Schizotypy Using the SIS

Possible schizotypal traits of the HR- and LR-adoptees were assessed at the initial phase of the data collection by using the “observed during interview” section of the SIS. This interview-based research instrument has been developed for evaluation of schizotypal signs and symptoms.^18^ The SIS is based on information acquired in a large Irish adoptive study and field-tested in 3 other locations. It is designed primarily for research purposes. The strength of this tool is that it takes into consideration subtle signs observed by the interviewer,^18,35^ who has been thoroughly trained in using it. These signs include 5 major groups of signs (rapport, affect, organization of speech, odd behavior, and suspiciousness) and several minor signs. In the current study, 27 items of schizotypal signs of the SIS were categorized into 4 subscales: negative, positive, and disorganized schizotypy, and general psychopathology. The signs and their categorizations to subscales are presented in the Appendix/Supplementary material. The symptoms were rated by the observer, from fair to very poor, from mild to marked, and from slightly to definitely odd and the scorings of each sign were dichotomized to indicate either presence or absence of a sign.

### Background Characteristics

Background characteristics were explored for both the HR- and LR-adoptees. These were gender (male or female), age (mean age), presence of psychiatric disorder (yes or no), educational level (no occupational studies, studies from work or courses, occupational studies, upper secondary school or high school studies or university studies), and marital status (unmarried, married or in a common-law relationship or widow or divorced), with each characteristic assessed at the initial phase of the study project. Adoptive family functioning measured using the GFRs was also included in the analyses. The background characteristics are presented in Table 1.

**Table 1.** Background Characteristics of Study Participants Having High (HR) or Low Genetic Risk (LR) for Schizophrenia Spectrum Disorders.

	High-risk (HR) adoptees (*n* = 127)	Low-risk (LR) adoptees (*n* = 130)	Test statistic^b^	Group difference, *P*-value
**Gender, *n* (%)** Male Female	55 (43.3) 72 (56.7)	64 (49.2) 66 (50.8)	*χ* ^2^(1) = 0.91	.341
**Age, mean (sd)**	27.9 (9.4)	27.1 (9.6)	t(255) = -0.731	.465
**Psychiatric diagnosis, *n* (%)** Yes No	37 (29.1) 90 (70.9)	30 (23.1) 100 (76.9)	*χ* ^2^(1) = 1.22	.269
**Educational level, *n* (%)** No occupational studies Studies from work or courses Occupational studies Upper secondary school/high school University No information	26 (20.5) 27 (21.3) 30 (23.6) 21 (16.5) 9 (7.1) 14 (11.0)	16 (12.3) 17 (13.1) 45 (34.6) 35 (26.9) 7 (5.4) 10 (7.7)	*χ* ^2^(5) = 12.04	.034
**Marital status, *n* (%)** Unmarried Married/in a common-law relationship Widow/divorced	57 (44.9) 62 (48.8) 8 (6.3)	64 (49.2) 57 (43.8) 9 (6.9)	*χ* ^2^(2) = 0.64	.727
**Global family rating, *n* (%)** ^a^ Functional family processes Mildly dysfunctional processes Dysfunctional processes	50 (42.7) 32 (27.4) 35 (29.9)	48 (38.1) 44 (34.9) 34 (27.0)	*χ* ^2^(2) = 1.62	.445

^a^Information missing for 14 study participants.

^b^Chi-square test for categorical variables, Student’s *t*-test for continuous variables.

### Global Family Ratings

The GFRs is a tool used for the evaluation of adoptive family functioning, relationships, and interaction.^30^ The GFRs include the following items: (1) anxiety, (2) boundaries, (3) parental coalition, (4) interaction and its quality, (5) flexibility of homeostasis, (6) transactional defenses, (7) conflicts, (8) empathy, (9) power relations, (10) reality testing, and (11) basic trust. The adoptive families were divided into categories from 1 (healthy families) to 5 (severely disturbed and chaotic families) based on the above-mentioned criteria. This classification, based on the Global Assessment of Relational Functioning (GARF), was initially published in DSM-IV (American Psychiatric Association, 1994). All interviews, tests, and observations of the GFRs were managed by researchers in the home environment when visiting adoptive families. All of these evaluations were performed with both individual family members as well as with different family coalitions.^30^ The researchers did not know the genetic risk status of the adoptees. The first 5 GFRs categories were re-grouped into the following 3 categories: (1) families with functional processes, (2) families with mildly dysfunctional processes, and (3) families with dysfunctional processes. Category 1 consists of the initial GFRs categories 1 and 2, category 2 consists of the initial category 3, and the new category 3 consists of the initial categories 4 and 5.

### Statistical Methods

The statistical significance of group differences in categorical variables was assessed with the Pearson’s chi-square test or Fisher’s exact test. The accuracy of the Strauss–Carpenter item mean score to separate adoptees by their genetic risk status for schizophrenia spectrum disorders was assessed using the area under the receiver operating characteristic curve (AUC-ROC). The better the measure of separability of the model is, the closer the value of AUC is to 1. Accordingly, when the measure of separability is poor, AUC is near 0. When AUC is 0.5, the model has no separation capacity. The statistical software used in the analyses was IBM SPSS statistics, version 25.

## Results

### Background Characteristics of the HR- and LR-adoptees

The background characteristics of the HR- and LR-adoptees are presented in Table 1. The only statistically significant difference between the HR- and LR-group was found in the educational level, where a greater proportion of HR-adoptees had no occupational studies or had lower-level education (ie, from work or courses).

### Real-word Functioning of HR- and LR-adoptees

As outlined in Table 2, no statistically significant differences were found between the HR- and LR-groups either in the domains or in any of the items in the SCLF-scale.

**Table 2.** Real-world Functioning Measured Using the Strauss–Carpenter Level of Functioning (SCLF)-scale Among the Adoptees at high (HR) or low (LR) risk for Schizophrenia Spectrum Disorders

Domains and items of domains (with range of scores in parenthesis) of the SCLF	High-risk (HR) adoptees (*n* = 127)	Low-risk (LR) adoptees (*n* = 130)	Test statistic^a^	Group difference, *P*-value
**Domain: function in past year (3 items)**				
Mean (sd) item score within domain	3.5 (0.5)	3.5 (0.6)	*t*(255) = -0.066	.947
Ability to meet own basic needs, *n* (%) Needs help (0–2) Needs no or a little help (3–4)	2 (1.6) 125 (98.4)	2 (1.5) 128 (98.5)	*χ* ^2^(1) = 0.00	.981
Fullness of life in past year, *n* (%) Relatively empty life (0–2) Relatively full life (3–4)	25 (20.2) 99 (79.8)	25 (19.5) 103 (80.5)	*χ* ^2^(1) = 0.02	.900
Overall level of function in past year, *n* (%) At least some impairment (0–2) No impairment or slight impairment (3–4)	16 (12.7) 110 (87.3)	18 (14.1) 110 (85.9)	*χ* ^2^(1) = 0.10	.750
**Domain: symptomatology (2 items)**				
Mean (sd) item score within domain	3.5 (0.5)	3.5 (0.5)	*t*(254) = 0.593	.554
Duration of non-hospitalization for psychiatric disorders during past year, *n* (%) 3 months or more (0–2) less than 3 months (3–4)	127 (100.0) 0 (0.0)	129 (100.0) 0 (0.0)	n.e.	n.e.
Absence of symptoms (in past month), *n* (%) Moderate to continuous signs and symptoms (0–2) No signs or symptoms (3–4)	39 (31.0) 87 (69.0)	36 (27.9) 93 (72.1)	*χ* ^2^(1) = 0.29	.594
**Domain: Social contacts (2 items)**				
Mean (sd) item score within domain	3.5 (0.7)	3.6 (0.6)	*t*(253) = 1.307	.192
Frequency of social contacts, *n* (%) Meets friends less than once a month (0–2) Meets friends at least once in 2 weeks (3–4)	11 (8.7) 115 (91.3)	5 (3.9) 124 (96.1)	*χ* ^2^(1) = 2.55	.110
Quality of social contacts in past year, *n* (%) Only moderately close or superficial relationships (0–2) At least one close relationship (3–4)	19 (15.1) 107 (84.9)	15 (11.6) 114 (88.4)	*χ* ^2^(1) = 0.66	.418
				
**Domain: Work (2 items)**				
Mean (sd) item score within domain	3.5 (0.5)	3.6 (0.5)	*t*(255) = 0.565	.573
Quantity of useful work in past year, *n* (%) Employed for less than ½ of the year’s working hours Or no useful work (0-2) Employed for at least ¾ of the year’s working hours (3-4)	4 (3.1) 123 (96.9)	7 (5.4) 123 (94.6)	*χ* ^2^(1) = 0.78	.376
Quality of useful work, *n* (%) Incompetent (0–2) Competent (3–4)	13 (10.3) 113 (89.7)	8 (6.2) 121 (93.8)	*χ* ^2^(1) = 1.43	.232
**Total Strauss–Carpenter scale (9 items)**				
Total scale, mean (sd) item score	3.5 (0.5)	3.5 (0.4)	*t*(255) = 0.733	.464
Total scale, sumscore, mean (sd; *n* = 350)	31.3 (4.1)	31.7 (4.0)	*t*(254) = 0.732	.476

Each item of the SCLF is dichotomized to indicate good (scores 3–4) or deteriorated (scores 0–2) functioning.

^a^Chi-square test for categorical variables, Student’s *t*-test for continuous variables.

Supplemental Table 1 shows the original distribution of each item (5-point rating scale, range 0–4) of the SCLF. The only statistically significant difference between study groups was found in the item “Ability to meet own basic needs,” where the LR-group was shown to need more help compared to the HR-group.

### ROC Analysis

**Figure 2.**
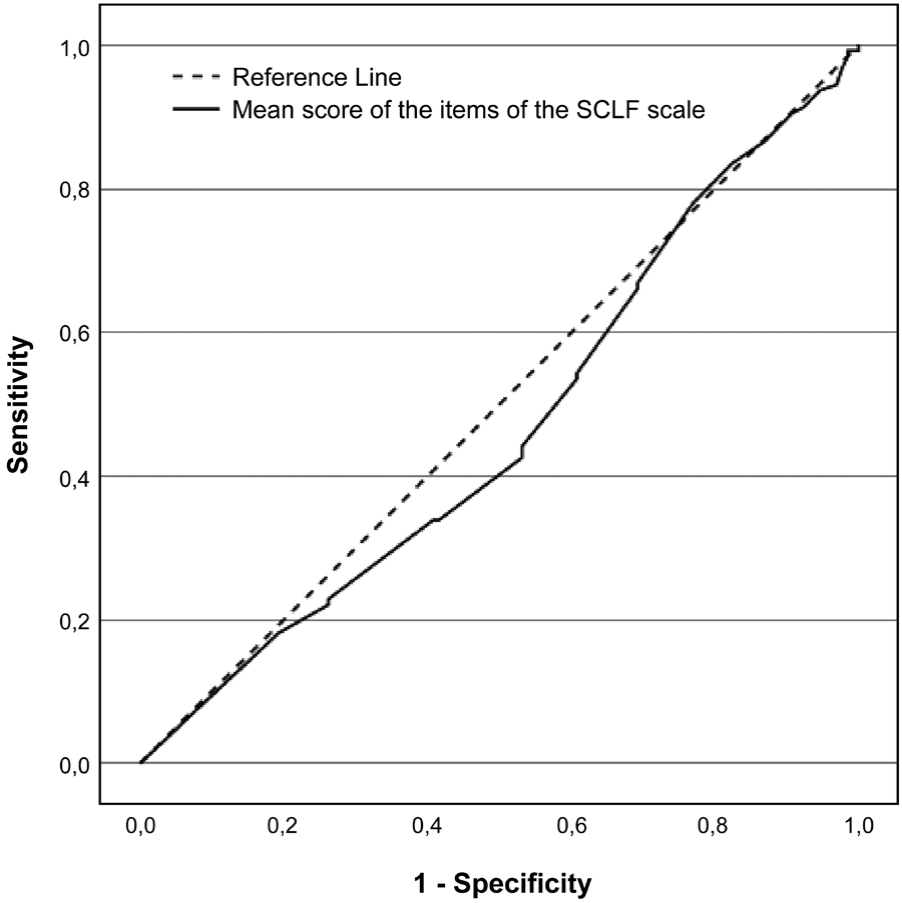
Receiver operating characteristics (ROC) curve for ability of overall level of functioning to identify adoptees with and without genetic liability for schizophrenia spectrum disorders.

The mean score of all SCLF items was utilized in ROC-analysis, to assess whether the level of functioning has any capability to distinguish the HR-adoptees from the LR-adoptees. Figure 2 shows that the adoptees’ level of functioning, as determined by the SCLF scale, did not differentiate them based on whether they were at high or low risk of developing schizophrenia spectrum disorders (AUC, area under analysis, AUC = 0.468, SE(AUC) = 0.036, 95 % CI for AUC 0.397–0.538).

### Level of Functioning in Relation to Schizotypy

**Figure 3.**
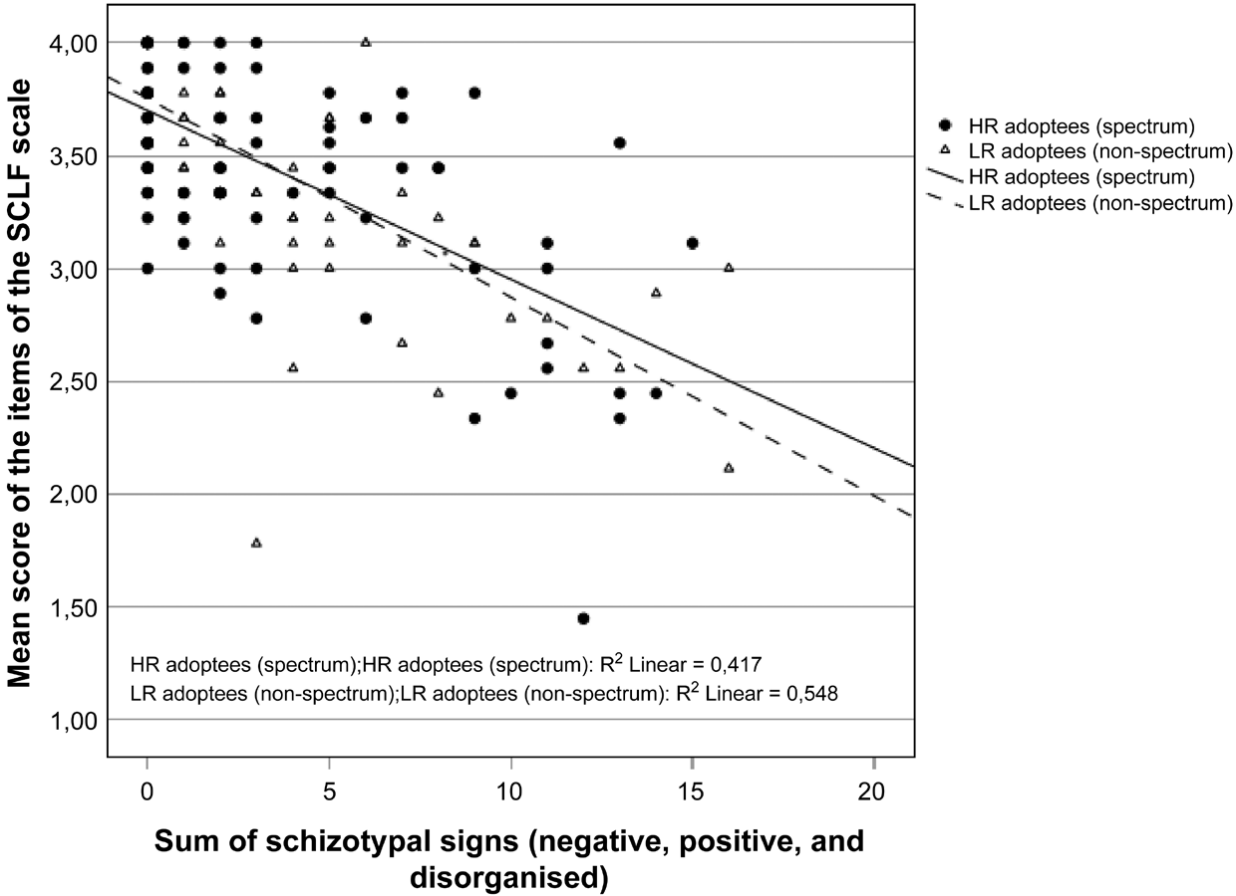
Comparison of the Prevalence of Schizotypal Traits and Level of Functioning of the HR- and LR-adoptees.

For additional analysis, the SIS was used to assess possible schizotypal traits among HR- and LR-adoptees. The SIS information was missing for a total of 49 adoptees. When comparing the prevalence of positive, negative, and disorganized schizotypal traits and general psychopathology between these groups (data not shown), no statistically significant differences were found between the study groups. Figure 3 shows the total amount of schizotypal traits in relation to the level of functioning of the HR- and LR-groups. In this figure, we can see a correlation between a higher number of schizotypal traits and lower scores in the SLOF.

## Discussion

Schizophrenia spectrum disorders are widely known to be associated with deficits in functioning. There is evidence that the same kind of deficiencies may also extend over generations to healthy probands. However, there is still some uncertainty regarding the contributions of genetic background and rearing environment to this association. In this study, we were able to compare structurally measured real-world functioning between genetically high (HR) and low (LR) risk groups for schizophrenia spectrum disorders in a set up with adoptees reared in a comparable adoptive family environments.

Our main finding was that there were no significant differences in either the total score of the Strauss–Carpenter Level of Functioning scale or in its all 4 domains, including overall functioning, symptomatology, social contacts, and work. This was contrary to our hypothesis, that the HR-population would have more deficiencies in global real-world functioning. Our hypothesis was based on findings of previous studies, proposing that this group has more deficiencies in executive functioning and cognitive performance.^9–11,15,24^

One plausible explanation for the difference between our findings and earlier studies is that the earlier studies have been performed on fundamentally more narrow and detailed areas of functioning with more sensitive measurements. However, direct comparisons are challenging, because there are no earlier studies of the comprehensive ability to function in everyday life among HR-populations. Another explanation for the difference in relation to earlier studies is that environmental effects could have caused the changes in functioning reported in them.^9,11,14,15^ Our study differed in that the rearing environments of the adoptees were comparable: the adoptive families were matched, and the comparability was confirmed by using GFR. However, it is possible that the scarce differences were at least to some extent due to the fact that the adoptive families of both groups were mainly well functioning and provided an adequate growth environment. HR-adoptees are especially sensitive to poor environments, but this tendency might not emerge at all in well-functioning families, because in previous research on the same material, it has been shown that a well-functioning family reduces thought- and psychiatric disorders in the HR-group.^29, 36^ This conclusion is also supported by the findings of the study of Li et al, where the genetic risk for schizophrenia, together with victimization for abuse, violence, and poor family dynamics was found to reduce executive functioning but victimization seemed to be the main predictor for this kind of impairments.^24^ Thus, it can be concluded that the genetic predisposition itself does not cause changes in overall real-world functioning and the role that environment has on one’s functioning is not as significant when negligence is not present.

Our findings demonstrate how crucial it is to take the rearing environment into account when comparing the many aspects of functioning across 2 genetically different groups. They also highlight the importance of support given from the environment to the high-risk groups.

In the background characteristics of our study, it was found that a notable proportion of high-risk adoptees had no formal education compared to the low-risk group. This was defined as having no occupational studies or having achieved studies from work or courses. Interpreted with caution, this finding could be explained by the possible connection between a high genetic risk for schizophrenia and the cognitive deficits shown in previous research.^15^ Conversely, this interpretation is not supported by our finding from the Strauss–Carpenter scale in the work domain, where no differences were found between the HR- and LR-groups in the quality or quantity of useful work. Consequently, it seems that individuals with a genetically HR for schizophrenia do not appear to have any reduction in their ability to perform tasks in their daily lives.

When reviewing the single items of functioning, an interesting and unexpected finding was that the HR-group seems to need a little less help in their own basic needs, such as feeding themselves and keeping clean, compared to the LR-group. Despite the finding being statistically significant, it must be interpreted with caution, because the difference between HR- and LR-groups only appeared in a relatively few study subjects. Further, the significance of the difference may also be reduced by the fact that there were no adoptees across samples who reported to need considerable or total help. However, a possible explanation for this difference could have been that individuals with genetic liability for schizophrenia may be less prone to express their needs and ask for help due to the emphasis of schizotypal features in this group. For example, social anxiety, which has been found to appear more commonly among persons with schizotypal features^22,32,37^ can impact an individual’s ability to ask for help.

In our study, we were able to compare the prevalence of different schizotypal features between HR- and LR-adoptees. The prevalence of schizotypal traits (negative, positive, and disorganized schizotypy and general psychopathology) did not differ between these groups. It is unlikely that the differences in ability to ask for help are related to schizotypy, since the HR- group does not seem to have more schizotypal traits, despite their genetic predisposition. In our study, the LR-group also had slightly more social contacts, and this might make it easier and more natural for them to ask for help. In addition to differences in personality features and social contacts, the difference in the need for help with basic needs might be explained by differences in physical conditions. However, we were not able to examine this area in our data and this possible association remains unconfirmed.

### Strengths and Limitations

The strength of this study is that we were able to measure real-world functioning using a structured scale, completed by trained and experienced interviewers. This provides a more objective and reliable assessment than self-completed questionnaires. The adoption setup makes it possible to control both the environmental features of the adoptive family and the genetic risk for schizophrenia spectrum disorders inherited from the biological mother. The psychiatric diagnosis of the study population according to DSM-III-criteria was defined using appropriate structured diagnostic methods by interviewing the adoptees and utilizing all available information from national health care registers.^26^ With regards to limitations, a weakness of the Strauss–Carpenter Level of Function scale is that it gives a general view of functioning and may not be able to differentiate possible subtle variations in different areas of functioning. Another limitation is that the nationwide study group is relatively small, making it possible that some findings could not be detected, and others might have been found by chance. A possible explanation for the main finding that needs to be considered in the limitations is that the 20 adoptees (HR *n* = 19, LR *n* = 19) who were later diagnosed with schizophrenia spectrum disorders were not included in the study. This exclusion was made because we needed to make sure that the disorder itself did not affect the adoptees’ functioning. It is probable that the HR-group would have been seen having poorer results in the SCLF, if these adoptees would also have been observed. Also, since no genetic analyses were conducted, polygenic scores for functioning, cognition, educational attainment, and psychiatric conditions remained unknown. According to previous research, comparable indirect methods, such as family studies have been shown to contribute important information besides genetic research.^38–40^ For example, The Social and Occupational Functioning Scale (SOFAS) and Global Assessment of Functioning (GAF) could be alternative functioning measures.^41–43^ There is also a 4-item version of the Strauss–Carpenter scale, which has shown to be appropriate for assessment of functioning in patients with bipolar disorder.^44^

### Future Research

For even more objective results about real-world functioning, using structured instruments where all different aspects of functional capacity are taken into consideration and evaluated by a professional is crucial. We also suggest assessment of functioning to be completed by an occupational therapist through behavioral observation, since information based on self-reported questionnaires can be limited.

## Conclusions

Our ability to compare structurally assessed real-world functioning between HR- and LR-adoptees with a similar growth environment has allowed us to gain fresh insights into the relationship between genetic vulnerability to schizophrenia and real-world functioning. In our study, it was not possible to distinguish HR-adoptees without schizophrenia spectrum illnesses from LR-adoptees based on their functioning. This suggests that possible functional differences between the HR- and LR-groups, seen in earlier studies with no controlling for the growth environment, are too subtle to manifest in real-world performance and are, in part, due to factors relating to the rearing environment. When assessing the functioning of 2 genetically different groups, consideration should be given to environmental factors because real-world performance does not reflect a person’s genetic risk for schizophrenia spectrum disorders. The impact that the environment has on functioning seems to be relevant mainly when the growth environment is not adequate.

## Supplementary Material

sgaf011_suppl_Supplementary_Table_S1
