# Supplementary material for: Differences in Real-world Functioning Between Adoptees With High or Low Risk for Schizophrenia Spectrum Disorders—The Finnish Adoptive Family Study of Schizophrenia
Source: Schizophr Bull Open. 2025 May 26;6(1):sgaf011. doi: 10.1093/schizbullopen/sgaf011 (PMC12243108; doi:10.1093/schizbullopen/sgaf011)
Supplement: sgaf011_suppl_Supplementary_Table_S1 [file sgaf011_suppl_supplementary_table_s1.docx]

SUPPLEMENTARY MATERIAL:

Supplemental table 1: Distribution of each item (five-point rating scale, range 0 - 4) of the Strauss-Carpenter Level of Function scale, by genetic risk status for schizophrenia spectrum disorder of the adoptees.

| **Strauss-Carpenter items with response options**  **and their scoring (in parenthesis)** | **High-risk (HR) adoptees (n=127)** | **Low-risk (LR) adoptees (n=130)** | **Test statistic^1^** | **group difference, p-value** |
| --- | --- | --- | --- | --- |
| Duration of non-hospitalization for psychiatric disorders in the past year, n (%)  no hospitalization (4)  less than 3 months (3)  3-6 months (2)  6-9 months (1)  more than 9 months (0) | 126 (99.2)  1 (0.8)  -  0 (0.0)  0 (0.0) | 126 (97.7)  3 (2.3)  0 (0.0)  0 (0.0)  0 (0.0) | n.a. | 0.622 |
| Frequency of social contacts, n (%) n (%)  Meets friends at least once a week (4)  Meets friends once every two weeks (3)  Meets friends once a month (2)  Does not meet friends except “over the back fence”  or at work or school (1)  Does not meet friends at all (0) | 91 (72.2)  24 (19.0)  8 (6.3)  (2.4)  0 (0.0) | 101 (78.3)  23 (17.8)  5 (3.9)  0 (0.0)  0 (0.0) | FFH = 3.81 | 0.277 |
| Quality of social contacts, n (%)  One or more close relationships (4)  One or more rather close relationships (3)  One or more moderately close relationships (2)  Only rather superficial relationships (1)  Only very superficial relationships (0) | 77 (61.1)  30 (23.8)  13 (10.3)  6 (4.8)  0 (0.0) | 82 (63.6)  32 (24.8)  12 (9.3)  3 (2.3)  0 (0.0) | FFH = 1.22 | 0.782 |
| Quantity of useful work, n (%)  Employed full-time continuously (4)  Employed for about ¾ of the year’s working hours (3)  Employed for about ½ of the year’s working hours (2)  Employed for about ¼ of the year’s working hours (1)  No useful work (0) | 115 (90.6)  8 (6.3)  2 (1.6)  1 (0.8)  1 (0.8) | 115 (88.5)  8 (6.2)  6 (4.6)  1 (0.8)  0 (0.0) | FFH = 3.07 | 0.638 |
| Quality of useful work, n (%)  Very competent (4)  Competent (3)  Moderately competent (2)  Marginally competent (1)  Incompetent (0) | 42 (33.3)  71 (56.3)  12 (9.5)  1 (0.8)  0 (0.0) | 49 (38.0)  72 (55.8)  8 (6.2)  0 (0.0)  0 (0.0) | FFH = 2.24 | 0.517 |
| Absence of symptoms (in past month), n (%)  No signs or symptoms (4)  Slights signs or symptoms most of the time or moderate signs  and symptoms on rare occasions (3)  Moderate signs and symptoms sometimes (2)  Severe signs and symptoms sometimes or moderate signs and  symptoms continuously (1)  Continuous and severe signs and symptoms (0) | 38 (30.2)  49 (38.9)  31 (24.6)  6 (4.8)  2 (1.6) | 45 (34.9)  48 (37.2)  29 (22.5)  6 (4.7)  1 (0.8) | FFH = 1.10 | 0.916 |
| Ability to meet own basic needs, n (%)  Needs no help (4)  Needs a little help (3)  Needs some help (2)  Needs considerable help (1)  Needs total help (0) | 119 (93.7)  6 (4.7)  2 (1.6)  0 (0.0)  0 (0.0) | 111 (85.4)  17 (13.1)  2 (1.5)  0 (0.0)  0 (0.0) | FFH = 5.62 | 0.047 |
| Fullness of life in past year, n (%)  Very full life (4)  Full life (3)  Moderately full life (2)  Relatively empty life (1)  Vegetative existence (0) | 34 (27.4)  65 (52.4)  20 (16.1)  5 (4.0)  0 (0.0) | 46 (35.9)  57 (44.5)  23 (18.0)  2 (1.6)  (0.0) | FFH = 3.69 | 0.302 |
| Overall level of function in past year, n (%)  No impairment (4)  Slight impairment most of the time or moderate  impairment rarely (3)  Moderate impairment sometimes (2)  Severe impairment sometimes of moderate  impairment continuously (1)  Continuous and severe impairment (0) | 73 (57.9)  37 (29.4)  12 (9.5)  4 (3.2)  0 (0.0) | 71 (55.5)  39 (30.5)  14 (10.9)  3 (2.3)  1 (0.8) | FFH = 1.37 | 0.951 |

^1^ Fisher-Freeman-Halton (FFH)Exact test for variables with three or more response options. n.a. = not available for Fisher Exact test with two response options.

| Supplemental table 2: Categorization of the Structured Interview for Schizotypy (SIS) (Kendler et al., 1989) items. | | | | | | | |
| --- | --- | --- | --- | --- | --- | --- | --- |
|  |  | **Items of SIS** | **Item values indicating presence of a trait** | **negative schizo-typy** | **positive schizo-typy** | **dis-organised schizo-typy** | **general psychopathology** |
| **Major sign** |  |  |  |  |  |  |  |
| Rapport | U1 | Eye contact | from less than average to absent | x |  |  |  |
|  | U2 | Body language | from fair to very poor | x |  |  |  |
|  | U3 | Emotional rapport | from fair to very poor | x |  |  |  |
|  | U4 | Global rapport | from fair to very poor | x |  |  |  |
| Fullness of affect | U5 | Fullness of affect | from fair to very poor | x |  |  |  |
|  | U6 | Appropriateness of affect | from fair to very poor |  | x |  |  |
|  | U7 | Lability of affect | from fair to very poor |  | x |  |  |
|  | U8 | Warmth of affect | cold or very cold |  | x |  |  |
| Organization of speech | U9 | Goal-directedness of thinking | from fair to very poor |  |  | x |  |
|  | U10 | Organization of associations | from fair to very poor |  |  | x |  |
|  | U11 | Rate of speech | slow or very slow |  |  |  | x |
|  | U12 | Amount of speech | definite or possible poverty of speech | x |  |  |  |
| Odd behaviour | U13 | Motor behaviour | from slightly to definitely odd |  | x |  |  |
|  | U14 | Social behaviour | from slightly to definitely odd |  | x |  |  |
|  | U15 | Odd dress | from fair to very poor |  |  |  | x |
|  | U16 | Global oddness | from fair to very poor |  | x |  |  |
| Suspiciousness | U17 | Nonverbal suspiciousness | from mild to marked |  | x |  |  |
|  | U18 | Verbal suspiciousness | from mild to marked |  | x |  |  |
| **Minor signs** |  |  |  |  |  |  |  |
|  | U19 | Irritability | from mild to marked |  | x |  |  |
|  | U20 | Mood | sad or very sad |  |  |  | x |
|  | U21 | Anxiety | somewhat or very anxious |  |  |  | x |
|  | U22 | Attention-seeking behaviour | moderate or marked |  |  |  | x |
|  | U23 | Flirtatious behaviour | moderate or marked |  |  |  | x |
|  | U24 | Self-centred behaviour and/or narcistic | moderate or marked |  |  |  | x |
|  | U26 | Social functioning | from fair to very poor | x |  |  |  |
|  | U27 | Psychiatric functioning | from fair to very poor |  |  |  | x |
|  |  |  |  |  |  |  |  |
| Kendler, K. S., Lieberman, J. A., & Walsh, D. (1989). The structured interview for schizotypy (SIS): A preliminary report. *Schizophrenia Bulletin, 15*(4), 559-571. doi:10.1093/schbul/15.4.559 | | | | | | | |
